# A Portable and Autonomous Magnetic Detection Platform for Biosensing

**DOI:** 10.3390/s90604119

**Published:** 2009-05-27

**Authors:** José Germano, Verónica C. Martins, Filipe A. Cardoso, Teresa M. Almeida, Leonel Sousa, Paulo P. Freitas, Moisés S. Piedade

**Affiliations:** 1 INESC-ID Instituto de Engenharia de Sistemas e Computadores-Investigação e Desenvolvimento, Rua Alves Redol, 9, 1000-029 Lisbon, Portugal; 2 INESC-MN Instituto de Engenharia de Sistemas e Computadores-Microsistemas e Nanotecnologias ans IN-Institute of Nanoscience and Nanotechnology and IN-Institute of Nanoscience and Nanotechnology, Rua Alves Redol, 9, 1000-029 Lisbon, Portugal; 3 Instituto Superior Técnico, TU Lisbon, Av. Rovisco Pais, 1049-001 Lisbon, Portugal

**Keywords:** biomolecular recognition detection, magnetoresistive sensors, magnetic nanoparticles, lab-on-chip, portable platform, digital signal processing

## Abstract

This paper presents a prototype of a platform for biomolecular recognition detection. The system is based on a magnetoresistive biochip that performs biorecognition assays by detecting magnetically tagged targets. All the electronic circuitry for addressing, driving and reading out signals from spin-valve or magnetic tunnel junctions sensors is implemented using off-the-shelf components. Taking advantage of digital signal processing techniques, the acquired signals are processed in real time and transmitted to a digital analyzer that enables the user to control and follow the experiment through a graphical user interface. The developed platform is portable and capable of operating autonomously for nearly eight hours. Experimental results show that the noise level of the described platform is one order of magnitude lower than the one presented by the previously used measurement set-up. Experimental results also show that this device is able to detect magnetic nanoparticles with a diameter of 250 nm at a concentration of about 40 fM. Finally, the biomolecular recognition detection capabilities of the platform are demonstrated by performing a hybridization assay using complementary and non-complementary probes and a magnetically tagged 20mer single stranded DNA target.

## 1. Introduction

Biochips are biological sensing devices used in lab-on-chip platforms to obtain higher levels of integration and, nowadays, are already used as disposable cartridges [1]. Recently, magnetoresistive (MR) biochips have been used for integrated biorecognition assays, using target biomolecules marked with magnetic particles (MPs) [2–6]. When compared to standard fluorescence techniques, MR biochips offer higher sensitivity, increased portability and lower cost [7]. The recognition assay consists on a biological reaction that allows the detection of *a priori* unknown biomolecules (e.g., human DNA strand for genetic disease detection or bacteria/cell detection).

**Figure 1.**
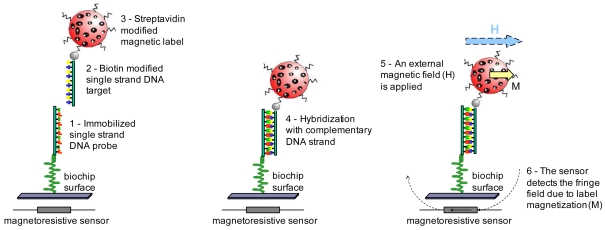
Detection of complementary single strand DNA.

Figure 1 depicts the DNA biorecognition steps when pre-labeled targets are used [3]. First a DNA strand with a known sequence (probe DNA) is immobilized on the biochip surface. The biorecognition assay starts with the introduction of an unknown DNA sample (target DNA) previously specifically labeled with a MP. This target DNA corresponds to the DNA to be analyzed. If the probe and target strands are complementary, a biomolecular reaction (hybridization) occurs binding the two strands together. A washing step removes all DNA strands which were not recognized. Finally, an external magnetic field is applied to magnetize the particles. The fringe field created by the immobilized MPs is detected by the MR sensor. The sensor's electrical resistance variation is proportional to the number of biomolecular recognition events.

The research in the pursuit of a fully integrated platform capable of performing biorecognition assays has experienced a major evolution in the last few years. The Philips Research Laboratories have developed a biochip that internally generates the magnetic excitation signal using on-chip aluminium tracks [8]. This solution was adopted in order to develop a compact detection platform, which incorporates the electronics required to drive MR spin-valve (SV) sensors and to perform signal conditioning. In that system, the acquired signals are processed and analyzed in a personal computer (PC). A complementary metal-oxide semiconductor (CMOS) integrated biochip based on SV sensors was also designed and implemented at Stanford University [9]. Using on-chip multiplexing techniques, the circuit is able to address 1,008 sensing sites. Although the analog front-end electronics are integrated in the CMOS biochip, external magnets are used to drive the sensors and an off-chip acquisition system is required to acquire the signals. The Naval Research Laboratory has continuously performed research in this field and has developed a biochip with 64 individually addressable giant magnetoresistance (GMR) sensors [10]. The magnetic sensor excitation signal is generated by a custom designed off-chip electromagnet integrated with a fluidics cartridge. Significant advances were also achieved at Brown University in the usage of magnetic tunnel junction (MTJ) sensors for biomolecular recognition detection [11].

This paper describes a fully autonomous and portable platform designed to incorporate all the electronics required to perform a biorecognition assay. The implemented platform supports linear and matrix type biochips using SV or MTJ sensors and is capable of addressing up to 256 sensing elements. Sensor signals are locally processed in real time in the platform and results are displayed in a PC or a personal digital assistant (PDA) through a graphical user interface (GUI) that allows full control over the assay.

The paper is organized as follows. Section 2. introduces the types of biochips supported by the platform and presents its most relevant electrical specifications. The proposed architecture and its most important circuits are introduced in Section 3., while at Section 4. the remaining circuits and the platform prototype are described. Experimental results showing the measurement noise level, magnetic nanoparticle detection and biomolecular recognition detection are presented in Section 5. Finally, Section 6. provides the paper's conclusions.

## 2. Magnetoresistive biochips: characteristics and specifications

**Figure 2.**
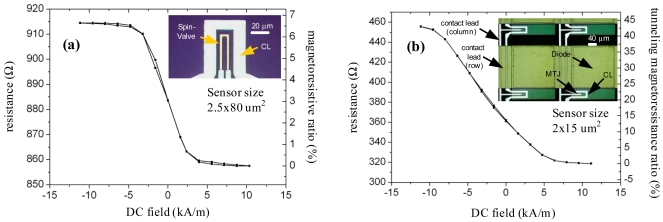
Microphotography and transfer curve of MRsensors of two biochip sensing sites: (a) spin-valve in a lineararray; and (b) magnetic tunnel junction in a matrix.

Magnetoresistive sensors are magnetic field transducers that, when properly biased, exhibit a linear change in electrical resistance under an external magnetic field [12]. Biochips with both SV and MTJ sensors were considered for the development of the platform presented in this paper. These sensors present a linear range of few kA/m that is controlled by the demagnetization factor of the sensor free layer [12]. For particle detection, the particles are excited by an AC magnetic field (field parallel to the sensor plane, transverse to the sensor length) and the sensor is biased by a DC, in plane, transverse magnetic field. The DC magnetic field biases the sensor to a specific point in the transfer curve in order to achieve maximum sensitivity. The more commonly used MR sensors for biochip applications are SVs, due to their high signal to noise ratio and their simpler fabrication process. Although, in some applications like the direct analysis of unamplified biological samples, an increased sensitivity is required which has led to research in biochips based on MTJs sensors [11, 13]. Several types of biochips have been developed at INESC-MN to target different objectives. Figure 2 shows transfer curves for properly biased U-shaped SV and MTJ sensors incorporated in a linear array (32 sensors) or a thin film diode/MTJ matrix (256 nodes). For both biochips, the sensing site includes a U-shaped current line (CL) that, in combination with an external transverse magnetic field, enables the magnetically labeled target DNA to be attracted towards the immobilized probes. This procedure is performed only to attract the labeled target DNA, the lines are not active during the remaining of the measurement procedure.

In the SV based biochip 32 SV sensors are organized in four different areas, each area with seven active sensing units and one reference sensor. The magnetoresistive ratio for these sensors is about 7%, its resistance is in the range of 700-1,200 Ω and require a bias current in the range of 850-1,000 *μ*A. For example, for 250 nm particles (Nanomag^®^-D, Micromod), using a DC bias field of 2.39 kA/m and an AC magnetizing field of 1.07 (kA/m)_rms_ (at 31 Hz), the sensor's output varies about 50 nV_rms_ for single particle detection. The starting sensor output is around 6 mV_rms_ (no particle present). Each sensing site includes a rectangular gold pad over the MR sensor (separated by a 0.3 *μ*m thick SiO_2_/Al_2_O_3_ passivation layer). The gold pad is used for biomolecular probe immobilization via thiol groups [14]. The U-shaped current line (CL) has a typical resistance of 90 Ω and a DC current of 40 mA is required to perform an effective MP attraction. The attraction procedure also requires an external DC magnetic field of 3.18 kA/m.

For high-throughput applications (i.e. genetic disease detection, genetic expression analysis) the biochip needs to comprise a larger number of sensors and thus a new type of biochip was designed, incorporating at each sensing site a MTJ sensor in series with a thin film diode, in a configuration similar to an architecture already employed for magnetic random access memory (MRAM) [15]. As in MRAMs, a transistor or a diode is used as a switching element. The elements are connected in a regular matrix organization and biochips with 256 (16×16) elements were already fabricated and tested [13]. Currently, the target specifications for these sensors are a tunneling magnetoresistance ratio (TMR) of 50% and a resistance in the range of 300-1,000 Ω. The bias current required to achieve the maximum sensitivity is in the range of 200-1,000 *μ*A. Current lines are also used in this architecture to attract the target DNA, see Figure 2(b). The required current is also 40 mA but its resistance is slightly higher, about 110Ω; the external DC field for the attraction procedure is 3.18 kA/m.

The noise level of the sensing elements depends on the biochip architecture and on the type of MR sensor. For a SV sensor with a resistance of 859 Ω, a noise of $60\;\text{nV}/\sqrt{\text{Hz}}$ is measured at 200 Hz, for a bias current of 1,000 *μ*A. On the other hand, a 319 Ω MTJ biased with 900 *μ*A shows a noise level of $105\;\text{nV}/\sqrt{\text{Hz}}$ at the same frequency [16]. In matrix type biochips, the noise level also depends on the switching element which may increase the total noise level of the sensing site [17]. The first step to develop a fully integrated electronic platform for biomolecular recognition detection was to perform the sensor modeling and characterization, in order to specify the measurement set-up, which has been achieved by the authors and for which results were published in previous papers [3, 16–18].

## 3. System architecture and circuit design

**Figure 3.**
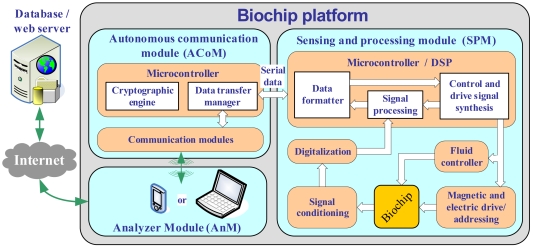
Architecture of the biochip platform.

To achieve an autonomous, flexible and secure system, the platform must also fulfill the following four requirements: *i)* perform real time signal processing; *ii)* use standard communication technologies to increase compatibility and interoperability; *iii)* use secure communication channels; and *iv)* provide a digital analyzer with a GUI, supported on a PDA or a PC, that allows full control of the measurement procedure. The measurement procedure defined in Section 1. and the requirements mentioned above, drives the definition of the platform architecture that is depicted in Figure 3.

The platform is composed by three main modules: *i)* a sensing and processing module (SPM); *ii)* an autonomous communication module (ACoM); and *iii)* an analyzer module (AnM). The SPM and the ACoM compose an embedded system which communicates with a more general computing device, such as a PC or a PDA. The AnM allows the control of the embedded system and also provides an internet access that can be used to store experimental data on a remote server.

### 3.1. Sensing and processing module

**Figure 4.**
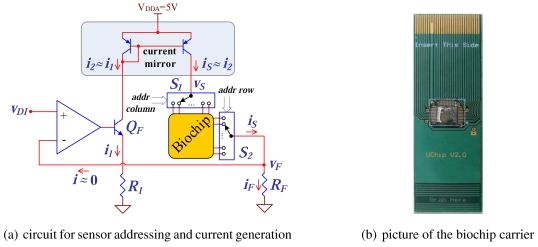
Biochip electric drive and interface.

The biochip requires electric and magnetic driving signals and also a fluid controller. The required signals are generated with the supervision of the microcontroller/digital signal processor (MC/DSP) through the control of digital to analog converters (DACs). The SPM includes a sensor interface block that performs multiplexing and conditioning of each sensor's signals. Figure 4(a) depicts the electric current generator for driving and biasing the biochip sensing sites and the circuit for addressing the biochip sensing sites [picture in Figure 4(b)]. Using a current controlled through a DAC output voltage ensures high flexibility as arbitrary waveforms can be generated (e.g., the synthesis of driving signals with a configurable frequency). The MC/DSP sets the amplitude of the driving current through a DAC. Signals can be synthesized using look-up tables or by calculating complex functions in the MC/DSP. The value of the current is defined by the DAC output voltage, *v_DI_*, and the resistor *R_F_*.

The DAC output voltage is converted to current by using a non-inverting amplifier topology with a NPN bipolar transistor (*Q_F_*) and a resistor (*R_F_*) in the feedback loop. Errors due the non-ideal behavior of the current mirror and the transistor are eliminated through this feedback loop that monitors *i_F_* through the voltage across *R_F_*. As the operational amplifier (OPAMP) input current is very low (nA range), the current passing through the biochip only depends of the DAC output voltage and the feedback resistor, *i_S_* = *i_F_* = *v_DI_/R_F_*. The generated current is multiplexed into the biochip allowing the usage of only a single current source for all the sensing sites. A sensing site is addressed by operating a sixteen channel demultiplexer (*S*_1_), to connect the programmable current source to a column of sites, and a sixteen channel multiplexer (*S*_2_), for addressing the row. The MC/DSP provides the row/column address of the site to be read, signals ***addr column*** and ***addr row***. The implemented prototype addresses up to 256 sensing sites for matrix type biochips, while for linear arrays this value drops to 30.

The magnetic drive circuit is one of the most critical parts of the measurement system. An AC magnetic field has to be applied, because the measurement is performed in AC in order to avoid low frequency noise. Since the optimal DC bias field is unknown, and depends on the type of MR sensor, the circuit must be able to generate positive and negative DC fields. A specific electromagnet must be designed for this specific application and targets the generation of the external magnetic field typical values provided in Section 2. The large size and required supply power make the optimization of the magnetic field generator a crucial aspect to deal with, in order to achieve a portable and autonomous platform. Besides having low size and power, because the noise generated by this circuit is added to the signals acquired from the sensor, it is necessary to guarantee that the generated noise has a very low level. The devised solution, described in Section 4., is based on an electromagnet and two low noise power OPAMPs.

Since the amplitude variation in the sensor's electric signal, caused by a single 250 nm MP, is in the order of tens of nanovolts, the noise introduced by hardware components of this block must be quite low. An additional requirement is low temperature sensitivity, because temperature can significantly change during recognition assay. The conditioned signals are then digitized and processed in the MC/DSP. A high resolution analog to digital converter (ADC) must be used in order to detect low concentrations of magnetic labels. Since 1 bit corresponds to 6.022 dB and $v_{AC}^{\text{sensor}}/v_{AC}^{\text{particle}}=6\times 10^{6}\;{\text{nV}}_{\text{rms}}/50\;{\text{nV}}_{\text{rms}}$ is about 100 dB (see Section 2.) a minimum resolution of 17 bits is required to measure $v_{AC}^{\text{particle}}$.

Most of the signal filtering and processing is performed in the digital domain, taking advantage of the processing capabilities provided by the MC/DSP. This simplifies the design and ensures a high degree of flexibility, since different algorithms may be implemented by simply changing the MC/DSP firmware. Finally, a data formatter converts the processed information into a format suitable to be serially communicated to the ACoM.

### 3.2. Autonomous communication module and analyzer module

In the core of the ACoM there is a data transfer manager and a set of standard interfaces for wire and wireless communication [6]. The transfer manager is responsible for communicating data and commands from and to the SPM and also for interfacing the autonomous communication module (ACoM) with the analyzer module (AnM). To be autonomous, the biochip platform has to be equipped with a battery. The ACoM monitors the state of the system battery and provides its recharge whenever it is necessary. The energy is drained from communication buses (e.g., universal serial bus (USB)), or alternatively from external power suppliers. Supply voltages required by the platform components are obtained by using low noise, high efficiency switched voltage converters. The cryptographic engine present in the ACoM assures privacy in the data transfer to the AnM. In the particular case of this application, a public-key or a symmetric cryptosystem can be applied [19]. Finally, the acquired data is transmitted to the AnM, using a standard communication interface (e.g., USB, Bluetooth and ZigBee).

The AnM is an autonomous computing system that has full control over the embedded system. The user interface allows the execution of complex preprogrammed tasks at the SPM. The AnM also acts as a second communication layer allowing an internet connection to a remote database by using WebServices. Using this connection the AnM can upload or download data from a database located in a remote server.

## 4. Platform prototype

**Figure 5.**
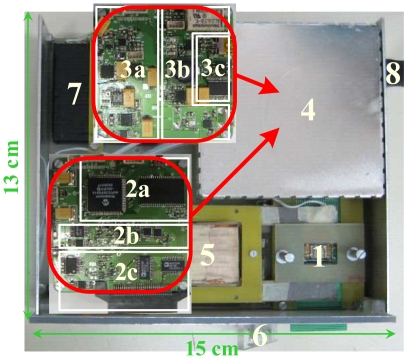
Topview of the platform prototype: 1) biochip encapsulated in a PCB chip carrier; 2a) digital signal controller and external memory; 2b) signal conditioning and acquisition; 2c) current generator, addressing circuits and biochip connector; 3a) magnetic drive; 3b) power supply circuits; 3c) USB interface; 4) noise shielding enclosure (the PCBs are placed inside this box); 5) electromagnet; 6) button to lift the electromagnet and allow the removal/insertion of the biochip; 7) battery; and 8) USB connector.

The developed portable platform prototype is depicted in Figure 5. Prototypes of the SPM and ACoM were implemented onto two printed circuit boards (PCBs) using off-the-shelf components. Both boards have equal size, only 32 cm^2^, and are stacked inside a steel noise shielding enclosure.

The full platform includes all hardware components that are required to perform a biorecognition assay and its size (13×15 cm^2^) ensures its portability. To attain low temperature drift and high precision in the measurements, thin film resistors were employed in the critical parts of the circuits. The autonomy of this design is also adequate as it can perform continuous recognition assays during eight hours when powered by a 3.7 V, 1,300 mA/h battery. The cryptographic engine and the fluid controller are still under development and are not included in the platform prototype herein described. The following subsections provide a description of the implemented circuits in which it is shown their most important characteristics. The AnM implementation for a PC is also presented.

### 4.1. Sensor addressing and drive signal generation

In the SPM, electric signals, required to drive the biochip, and to individually address and readout signals from individual sensing sites, are generated by a dsPIC30F6014 digital signal controller (DSC) [Figure 5(2a)]. This DSC is capable of performing up to 30 million instructions per second (MIPS) and it includes all the peripherals required to control the on-board electronic components. The SPM board also contains a 1 Mbit memory for storing acquired and processed data. Since the typical bias current for SV or MTJ sensors is near 1 mA and the maximum DAC voltage output was chosen to be 2 V, *R_F_* was set to 1.74 kΩ [circuit depicted in Figure 4(a)]. After calibration, the generator error is less than 0.13%, for a current range from 144 *μ*A to 1.16 mA, and is mostly due to the integral non-linearity (INL) error of the DAC.

An important characteristic of the measurement system is the adoption of a PCB as chip carrier [Figure 4(b) and Figure 5(1)]. The biochip is wirebounded to the PCB which has an edge that is inserted into a connector placed in the SPM main board [Figure 5(2c)] that is placed inside the noise shielding enclosure [Figure 5(4)]. This allows an easy insertion of the biochip from outside the noise shielding enclosure where the SPM is located. Another advantage is the possibility to perform signal routing in the chip carrier. This ensures compatibility with different biochips and allows the design to support both matrix and linear array types like the ones in [13, 20]. Furthermore, it is easier to integrate it with the microfluidic transport system that is being developed. The size of the carrier was also chosen to be compatible with automatic DNA spotters for probe arraying. Finally, a PCB chip carrier is less expensive than a conventional ceramic chip carrier, which is an important aspect since the biochip is intended to be disposable. A custom off-chip electromagnet is used to generate the required external magnetic excitation signal [Figure 5(5)]. It is worth to notice that the size of the electromagnet air gap is determined by the size of the biochip itself. Since the biochips are typically 1 cm wide, the electromagnet's total size must be significantly larger to ensure a proper operation. The circuit corresponding to the magnetic field generator was designed in order to give special attention to the low noise requirement in detriment of power efficiency.

**Figure 6.**
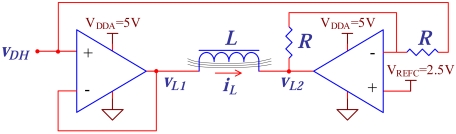
Magnetic drive circuit diagram.

The bridge topology in the circuit of Figure 6 ensures that the current, *i_L_*, flowing through the inductor *L* can be positive or negative, and doubles the maximum amplitude of the generated current when compared to a solution with a single OPAMP. The current is defined by a DAC output voltage, *v_DH_*, and by the inductor impedance, *i_L_* = (*v*_*L*1_ − *v*_*L*1_)/*Z_L_* = 2(*v_DH_* − *v_REFC_*)/*Z_L_*. Again, the usage of a DAC output voltage to control a signal generator ensures high flexibility The selected OPAMP (OPA567) can deliver a current up to 2 A and has low noise and low offset drift due to temperature. Furthermore, this circuit only requires a single power supply rail and a reference voltage, simplifying the design of the power supply network. The magnetic field generated by this circuit is limited by the saturation of the inductor core. The core was built using oriented grain steel foils (0.25 mm thick) to reduce power losses due to induced currents. Since size was one of the optimization targets, the inductor core section was reduced to the minimum area that does not lead to significant saturation. The designed electromagnet has a size of 65×45 mm^2^ and a core with a section of 15×6 mm^2^. A coil with 800 turns was winded on the core to achieve the required magnetic field, resulting in a coil inductance of 33 mH and a resistance of 5 Ω. At the frequency of 211 Hz, this circuit can generate a maximum AC magnetic field of 1.19 (kA/m)_rms_ with a DC superimposed that ranges from -3.18 kA/m to 3.18 kA/m. When the AC component is not required, it can generate a DC from -15.9 kA/m to 15.9 kA/m. This circuit is suitable to generate the magnetic field required both for determining the transfer curves of the sensors and for the biorecognition assay itself. The core transfer curve was measured and was used to build a calibration table. The table allows to correct the generated DC magnetic field. The AC magnetic field was calibrated using a sense coil. After calibration, the AC amplitude error, at a frequency of 211 Hz, is less than 0.2% for a DC range between -3.18 kA/m to 3.18 kA/m, and is mostly due to the nonlinear behavior of the electromagnet core.

The biochip also includes the CL to generate on-chip magnetic fields that guide the labeled biomolecules over immobilized biological probes. The current required for CLs is controlled using a pulse width modulation (PWM) signal generated by the DSC. Only two additional components are required: a transistor, operating as a switch and a resistor to limit the peak current. The prototype includes two CL drivers that can deliver up to 50 mA DC current to CLs with a resistance up to 100 Ω.

### 4.2. Signal conditioning and acquisition

**Figure 7.**
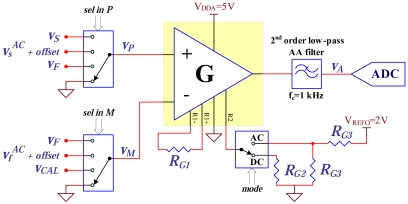
Signal conditioning and acquisition.

As depicted in Figure 7, the acquisition circuit is capable of performing AC and DC measurements for several different signals [picture in Figure 5(2b)]. The sensor signal is acquired using a single instrumentation amplifier (INA327) and a high resolution sigma delta ADC (LTC2440). A second order low pass anti-aliasing (AA) passive filter with a 1 kHz cutoff frequency is located at the output of the amplifier to eliminate high frequency signals. From the amplifier datasheet, for a gain of 40, its input referred noise level is $53\;\text{nV}/\sqrt{\text{Hz}}$ (*f* > 10 Hz). By using thin film resistors for setting the amplifier gain, it is possible to achieve a gain drift due to temperature of only 30 ppm/°C. The ADC resolution depends on the required conversion speed, with a 17 bit effective resolution assured for a 3.52 kHz conversion rate. The low noise and low drift of the amplifier along with the high resolution of the ADC make these components compatible with the detection of low concentrations of MPs.

The amplifier gain is set by the DSC through the control of a switch (signal ***mode*** in Figure 7), having a gain of 40 in AC mode and 1.2 in DC mode. When operating in AC mode, it is required to add an offset to the amplifier output to avoid signals below the signal ground level. The ***mode*** signal also selects the offset output voltage of the amplifier (0 V or 1 V). The DSC, using the signals ***sel in P*** and ***sel in M***, operates two switches located at the instrumentation amplifier input to allow the measurement of: the sensor voltage (*v_S_*) or only its AC component $\left(v_{s}^{AC}\right)$, the current generator feedback voltage (*v_F_*) or differential signals, namely (*v_S_* − *v_F_*) and $\left(v_{s}^{AC}-v_{f}^{AC}\right)$. Other signals are also available at the switches inputs to be used in the calibration procedure, namely the circuit ground and the DAC output voltage (*v_cal_*). A calibration procedure was defined to correct the gain value and offset errors. The OPAMP and ADC offset voltages are experimentally determined by setting both the amplifier inputs to ground. Since *v_F_* is equal to *v_DI_* in Figure 4(a), that is the output of a DAC, its measurement can be used to correct gain of the amplifier in both modes of operation. The resistance of the multiplexer and the demultiplexer, *S*_1_ and *S*_2_ in Figure 4, is determined with a procedure in which the biochip is replaced by a set of known precision metal film resistors. The difference between the measured resistance and the real value of the precision resistors is the sum of the multiplexer and the demultiplexer resistances. The calibrated circuit is able to measure resistance values with a maximum error of 0.15%.

### 4.3. Autonomous communication module and user interface

A simplified version of the ACoM presented in Section 3. was used in the work herein presented. This ACoM prototype only includes an USB interface [Figure 5(3c)], a battery charger and power supply circuits [Figure 5(3b)]. The ACoM provides two low noise power rails, set to 5 V, capable of delivering up to 500 mA each. During experiments the ACoM automatically disconnects the external power supply and the platform runs only on battery power. This procedure avoids the noise induced by the external power supply. The adopted USB converter provides standard UART signals and also status and control signals that allow the microcontroller to monitor the USB connection status and to control the battery charging current. To attain the SPM low noise requirements all data and control signals are electrically isolated from the remaining signals of the board by using optocouplers.

**Figure 8.**
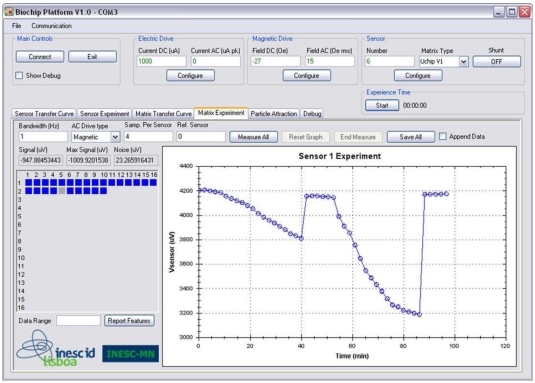
Snapshot of the graphical user interface for particle measurement; sensor biased with 1 mA, magnetic drive set to 2.15 kA/m DC and 1.19 (kA/m)_rms_ AC at 211 Hz, signal acquired at a sampling rate of 844 samples/s with a gain of 40 and the SPM digital bandpass filter set to 1 Hz [22].

Microsoft operating systems and tools were adopted to program and operate the AnM. Different classes were programmed in C# for decryption, communication and general user interfacing with the Microsoft Visual Studio .NET 2005 environment. Code portability is ensured by using the .NET 2.0 Compact Framework, which is supported by a broad range of Microsoft operating systems, both for mobile and desktop devices [21]. The developed GUI, programmed on a PC based AnM, allows configuring platform operation, including: the signal levels that are used to drive the sensors, the choice of biochip type, the address of the site to sense and the selection of different types of measurements to be performed. For example, a method is provided to acquire and store on the AnM the transfer curves for all biochip sensors. These measurements are then used to evaluate the behavior of the biochip sensors. The GUI for the particle detection measurement procedure, is depicted in Figure 8. Using this GUI, the user can configure the measurement parameters and can also select which sensors will be used in the signal measurement (the selected sensors are represented by blue squares). The results plotted in Figure 8 were obtained for two sequential measures of 250 nm MPs without DNA targets in two different concentrations, 81 pM and 8.1 pM, using a SV sensor with a resistance of 735 Ω and a magnetoresistive ratio of 7.44%.

At the user interface, the transmitted data is displayed in real time allowing the user to follow the measurement evolution. The screenshot shows the several phases that are required to perform particle detection. A first baseline was measured during 10 minutes, after this the suspension at 8.1 pM was dispensed on the biochip and was let to settle down during 30 minutes. After this the biochip was washed and the signal returned to the baseline. The number of particles above the sensor is proportional to the voltage difference between the baseline and the signal just before the washing step. The same procedure was applied to analyze the 81 pM suspension. The graphic shows a signal variation of about 0.4 mV_rms_ for the particle concentration of 8.1 pM and 1 mV_rms_ for the concentration of 81 pM, at the concentration of 81 pM the sensor is already saturated. The GUI can also display in real time a simple analysis of the measured data, such as the sensor's signal ( Signal (uV)), the maximum difference between the acquired samples ( Max Signal (uV)) and the noise level ( Noise (uV)). A summary of the experiment containing these data features for all the sensors in the biochip is calculated and displayed to the user by clicking on Report Features. The acquired data can also be stored, in extensible markup language (XML) or comma separated values (CSV) file format by clicking on the Save All button. This allows a detailed analysis of the measured data using more powerful software applications and provides a flexible way to store the acquired data.

## 5. Experimental results

The following subsections present results of the platform performance at different levels: measurement noise level, nanoparticle detection and biomolecular recognition detection.

### 5.1. Noise analysis

**Figure 9.**
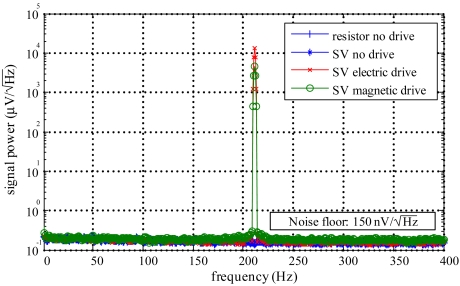
Power spectrum of the acquired signals for different loads and driving signals.

The total noise in the measurement system was experimentally evaluated for a biochip based on SV sensors and for a test load in which the biochip was replaced with a set of precision metal film resistors. The prototype boards were placed inside a steel box in order to block interference from external noise sources [Figure 5(4)]. The SVs average resistance is 735 Ω for a DC field of 1.99 kA/m and the metal resistors have a nominal value of 1 kΩ. All the measurements were performed using a 1 mA bias current and a DC field of 1.99 kA/m. Four different tests were performed in which the driving signals and the measured load were changed, namely the signal was measured in the following conditions: *i)* resistor load without AC drive; *ii)* SV load without AC drive; *iii)* SV load with an AC current drive of 65 *μ*A pp amplitude; and *iv)* SV load with a magnetic drive of 1.19 (kA/m)_rms_ AC amplitude. A 211 Hz sine wave with 16 quantization levels per period, based on a look-up table stored in the DSC internal memory, was synthesized. The signals were acquired with the amplifier configured in AC mode with a gain of 40 and was digitized at a rate of 844 samples/s. The signal's power spectrum for the different conditions is displayed in Figure 9.

Notice that since bins are used to calculate the signal's power spectrum, the energy associated with a carrier signal can have effect in multiple points of the spectrum. The SV signals with electric/magnetic drive are overlapped and have a large peak of 99 dB and 88 dB above the noise floor, respectively; without drive, no signal is noticeable. The signal level with the magnetic drive is lower due to the maximum allowed AC field and due to the fact that the sensor is used not far from the saturation region for the applied DC field. Analyzing the results one can conclude that the external interference was successfully blocked as the only visible peak occurs at the excitation frequency. No signal is noticeable at the power line frequency or at its harmonics. The noise floor is similar in all measurements and only in the magnetic drive a slight increase of about $30\;\text{nV}/\sqrt{\text{Hz}}$ was observed. This increase is due to the fact that the magnetic drive circuit represents an additional noise source which also contributes to the system's noise floor. According to the ADC datasheet, for the required sampling frequency, an effective resolution of 19 bits is achieved and the noise level contribution, referred to the input of the instrumentation amplifier, is $3\;\text{nV}/\sqrt{\text{Hz}}$. The noise floor level observed in Figure 9 is not surprising since, for the configured gain of 40, the amplifier alone has an input referred noise of $53\;\text{nV}/\sqrt{\text{Hz}}$. The acquired signal includes not only this noise source but also the noise introduced by the circuits used to generate the magnetic and electric driving signals and by all the other components of the platform. When using an AC driving signal, it can also be noticed an increase of the noise level around the driving signal's frequency. Finally, the signal range available for the measurements is almost 100 dB, which is suitable even for the detection of low concentrations of MPs.

### 5.2. Nanoparticle detection

The MP detection is performed by measuring the resistance variation of the MR sensor. Since the sensor bias current is known, the resistance can be determined measuring the *v_A_* voltage (see Figure 7). This voltage depends on several variables, namely: the amplifier gain *G*; the multiplexer series resistance *R_S_*_1_; the demultiplexer series resistance *R_S_*_2_; the DC component of the sensor resistance, $R_{S}^{DC}$, that only depends of the sensed DC magnetic field, {*H^DC^*}; and the AC component of the sensor resistance, $r_{s}^{AC}$, that depends of both the DC and AC component of the sensed magnetic field, {*H^DC^*, *H^AC^*}. Using these variables, when the amplifier is in AC mode and is set to measure the differential voltage *v_S_* − *v_F_*, the *v_A_* voltage can be defined as follows:

(1) $$v_{A}=G(v_{S}-v_{F})=i_{F}G(R_{S1}+R_{S2}+R_{S}^{DC}\{H^{DC}\}+r_{s}^{AC}\{H^{DC},H^{AC}\})$$

In our previous work, the sensor resistance was evaluated by only using DC measurements or AC measurements with the AC component introduced by current. In the results herein presented, the sensor resistance is determined using an AC magnetic drive. When an AC magnetic drive is applied and the amplifier is in AC mode, the value of the acquired voltage is $v_{A}=I_{F}Gr_{s}^{AC}\{H^{DC},H^{AC}\}$, since only the signal caused by the variation of the sensor resistance due to the applied AC magnetic field has an AC component. When compared to the measurement in DC, the noise component of the measured signal and the required dynamic range are both reduced. Since the measurement is performed in AC, the sensor resistance can be found by computing the discrete Fourier transform (DFT) of the acquired signal in the DSC [22]. To identify the signal component due to the MPs, the value of the sensor resistance is measured with and without MPs. This is achieved as follows: *i)* first the baseline voltage $\left(v_{\text{base}}^{AC}\right)$ is measured without MPs; *ii)* in the following step the MPs are placed on the top of the biochip; and *iii)* after waiting for the MPs to settle down, the signal is again evaluated $\left(v_{\text{sat}}^{AC}\right)$. The number of MPs above the sensor is proportional to $v_{\text{mps}}^{AC}=v_{\text{base}}^{AC}-v_{\text{sat}}^{AC}$.

**Figure 10.**
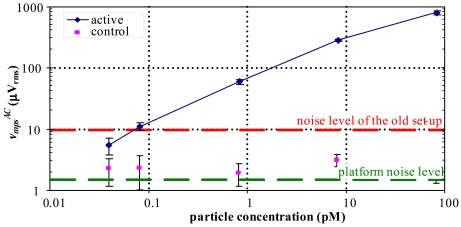
Particle signal, $v_{\text{mps}}^{AC}$, for several concentrations of MPs. Nineteen active and five control sensors were used, the length of the error bars corresponds to the standard deviations between the particle signals from different sensors.

To illustrate the measurement capabilities of the developed system, four MPs suspensions were prepared by seriated dilutions of 10× from an initial MP solution with 5 × 10^11^ particles/*μ*L in a phosphate buffer. A fifth suspension was prepared by diluting the stock suspension 20,000×. The tests were performed using MPs with 250 nm in diameter (Nanomag^®^-D, Micromod). The biochip used in these experiments is composed by 25 SV sensors with a parallel state resistance of 735±12 Ω and a magnetoresistive ratio of 7.44±0.03% (chip architecture in [20]). The sensors were biased with a1mADC current, the magnetic drive was set to 2.15 kA/m DC and 1.19 (kA/m)_rms_ AC at 211 Hz. In the experiments the sensors were sequentially addressed and the signal acquired using a gain of 40 at a sample rate of 844 samples/s. The acquired signal was then filtered with a 1 Hz bandwidth bandpass filter by using the algorithm described in [22], and the measurements were performed starting from the least to most concentrated suspension. Three steps are performed to analyze a suspension: *i)* wait 10 minutes for a baseline; *ii)* drop 10 *μ*L of the suspension onto the sensors and wait for 30 minutes; *iii)* wash the biochip using a phosphate buffer. Post-processing was performed in the AnM to compensate the temperature related signal variation. This post-processing was made by considering that the baseline before and after washing the sensors remains constant. This assumption is valid since the drive signals are constant and during baseline measurement no particles exist on top of the sensor and after the wash all the particles are removed and thereby do not cause variations in the magnetic field sensed by the MR sensor. Thus, it is possible to perform a linear regression in both regions. Using the average slope value of these two regressions, a correction line is determined. Finally, compensation is performed by subtracting the correction line to the acquired data. The corrected data from all sensors is displayed in Figure 10, where the curve represents the average values and the length of the error bars corresponds to the standard deviation. Five sensors were covered with silicone gel to prevent particle detection and are used as controls. The described compensation procedure reduced the average signal from the control sensors in about 50%.

As expected, an increase of $v_{\text{mps}}^{AC}$ with the particle concentration is observed. The lower value of particle concentration, 41 fM, correspond to a sensor area coverage of 2% (65 particles above the sensor). To allow a relative performance evaluation of the proposed platform, Figure 10 also shows the noise level of the measurement set-up in [20]. As it can be observed, the noise level of our platform is about one order of magnitude lower, and the error bars that represent the standard deviation are much smaller.

### 5.3. Biomolecular recognition detection

A model biomolecular assay was performed using 20mer single stranded DNA oligonucleotides hybridization. The DNA oligonucleotides were synthesized by MWG-Biotech (Ebersberg, Germany), encoding for a sequence of the conservative region of the 16S rDNA from *Escherichia coli*. Their designation, base sequences and modifications are as follows: complementary probe 5′ SH - TTT TTT TTT TTT TTT ACA CGG TCC AGA CTC CTA CG- 3′; non-complementary probe, 5′ SH - TTT TTT TTT TTT TTT CCT TAC CTG AAG GCT CCA CT - 3′; and complementary target, 5′ biotin - CGT AGG AGT CTG GAC CGT GT - 3′. The adopted surface chemistry consisted on the covalent immobilization of the thiol-modified (-SH group) probe biomolecule to the gold pad over the sensing area. Before probe immobilization the gold surface is submitted to a cleaning procedure including, a rinsing step with isopropanol and deionized water, and 15 min exposure to an ultraviolet light/ozone plasma inside an UVO cleaner (Jelight, USA). Immediately after, a 2 *μ*L of different (complementary and non-complementary) probe solutions at 1 *μ*M in TE-buffer (10 mM TRIS, 1 mM EDTA, 0.1 M K_2_HPO_4_, pH 7.4 corrected with HCl) are manually spotted at each one of the two separated biorecognition zones. Immobilization proceeds for 2 hours at 37°C. The unbound probe molecules are washed away using the same TE-buffer. The free gold surface is then blocked with a solution of 1 mg/mL of thiolated polyethyleneglycol (SH-PEG) in TE-buffer for 1 hour at 37°C. The target DNA is magnetically labeled by incubating biotinylated DNA targets with streptavidin-functionalized 250 nm MPs (Nanomag^®^-D, Micromod). The suspension was prepared by combining 2.5 *μ*L of the stock suspension (4.9×10^11^ particles/mL) with 100 *μ*L of target DNA at 1 *μ*M in PB/Tween buffer (phosphate buffer 0.1 M, pH 7.4, 0.02% (v/v) Tween20). After 30 minutes of reaction, the MPs carrying the target molecules are concentrated using a permanent magnet in a 20 *μ*L final volume.

The biochip used in these experiments is composed by 32 U-shape SV sensors with a parallel state resistance of 662±47 Ω and a magnetoresistive ratio of 5.89±0.03%. The sensors were biased with a 1 mA DC current and the magnetic drive was set to 2.79 kA/m DC and 1.07 (kA/m)_rms_ AC at 211 Hz. The CLs are used to attract the MPs target DNA conjugates to the sensor region. The CLs were driven with a 40 mA DC current and an external 0.2 Hz magnetic field with 2.39 kA/m_rms_ amplitude was applied. As in the MP detection experiment previously described, the sensors were sequentially addressed and the signal was measured using the same gain, sample rate and filtering procedure. Four steps are performed to analyze a suspension: *i)* acquire a 20 minutes baseline $\left(v_{\text{base}}^{AC}\right)$; *ii)* dispense 5 *μ*L of streptavidin coated MPs suspension per active biorecognition zone, stop the sensor signal acquisition, start the attraction procedure and wait 15 minutes; *iii)* stop the attraction, restart the signal acquisition and wait for 15 minutes to allow DNA hybridization; *iv)* wash the biochip by flushing with PB/Tween using a micropipette, while the sensor signal is continuously measured for another 15 minutes $\left(v_{\text{bind}}^{AC}\right)$. The number of recognition events above each sensor is proportional to $v_{\text{recog}}^{AC}=v_{\text{base}}^{AC}-v_{\text{bind}}^{AC}$.

**Figure 11.**
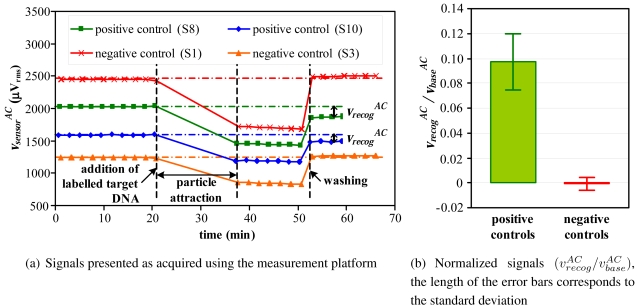
Magnetic detection of DNA-DNA biomolecular recognition using the portable platform.

Figure 11(a) presents untreated sensor responses acquired sequentially from four different sensors in the same chip, two of them measuring the complementary binding signals (positive controls) and another two referring to non-complementary signals (negative controls). After performing the postprocessing described in Section 5.2. to compensate temperature related signal variations the acquired data was analyzed. Notice that, although in this assay particles may remain above the sensor after the wash, they are immobilized which leads to a stable sensor voltage output. This makes the correction procedure previously described valid for this type of assay. The sensing curves corresponding to non-complementary DNA probe sequences, as expected, returned back approximately to the baseline level with an average $v_{\text{recog}}^{AC}$ signal of -2±12 *μ*V_rms_ for five sensors, meaning that no molecular recognition occurred and the background signal from non-specific interactions is small. The minus signal of this average value is due to temperature drift of the measurement system. On the other hand, the sensing curves corresponding to five probes complementary to the target molecule, after two washing steps, present a $v_{\text{recog}}^{AC}$ signal about 180±47 *μ*V_rms_. In Figure 11(b) the previous $v_{\text{recog}}^{AC}$ were normalized to $v_{\text{base}}^{AC}$. These results show that the system is able to identify and clearly distinguish the positive and negative controls.

## 6. Conclusions

The described portable platform is able to effectively replace a standard benchtop, bulky measurement apparatus as a much more practical and cost effective solution for biomolecular recognition detection. The presented platform prototype has a size of only 15×13 cm^2^ and includes almost all the modules that are required to perform a full biorecognition assay. It also has the ability to run only on battery power for almost eight hours, which enables its usage in field experiments where no external supply power is available. Furthermore, the platform is able to achieve even lower noise levels than a conventional setup, allowing the robust detection of smaller concentrations of MPs. It has been shown that the system is able to detect 250 nm MPs at 41 fM. Considering a target DNA functionalization protocol of 1 DNA strand per streptavidin molecule on the surface of the label [20], this limit corresponds to a DNA concentration of about 20 pM. Finally, a biorecognition detection assay has demonstrated the capabilities of the platform to clearly discriminate the signal from complementary and non-complementary controls. Additional biorecognition assays have been performed which showed that the developed platform achieves a femtomolar limit of detection [23].

Currently the remaining modules of the biochip platform, namely the cryptographic engine and the fluid controller, are in the prototyping stage. Additionally, a new method is being developed to improve the correction of the temperature related signal variations.
